# The relationship between multimorbidity and cognitive function in older Chinese adults: based on propensity score matching

**DOI:** 10.3389/fpubh.2024.1422000

**Published:** 2024-09-12

**Authors:** Yumeng Zhang, Xiaoli Yuan, Zhixia Jiang, Rujun Hu, Heting Liang, Qingyun Mao, Yan Xiong, Jiabi Zhang, Mi Liu

**Affiliations:** ^1^Department of Nursing, Affiliated Hospital of Zunyi Medical University, Zunyi, Guizhou, China; ^2^Faculty of Nursing, Zunyi Medical University, Zunyi, Guizhou, China; ^3^College Office, Guizhou Nursing Vocational College, Guiyang, Guizhou, China; ^4^Kweichow Moutai Hospital, Renhuai, Guizhou, China

**Keywords:** cognitive, multimorbidity, older adults, cognitive decline, propensity score matching

## Abstract

**Objective:**

The goal of this study was to further validate the effect of multimorbidity on cognitive performance in older adults after controlling for confounders using propensity score matching (PSM).

**Methods:**

A cross-sectional survey of older adult people aged 60 years or older selected by convenience sampling was conducted in seven medical institutions, three communities, and five nursing homes in Zunyi City, Guizhou Province. The data collected included general information, health-related information, and Mini-Mental State Examination (MMSE) scores. Variables were controlled for confounders by PSM to analyze differences in cognitive ability between multimorbidity and nonmultimorbidity older adults. Logistic regression and multivariate-adjusted restricted cubic spline (RCS) curves for matched samples were used to assess the relationship between multimorbidity and cognitive decline.

**Results:**

A total of 14,175 respondents were enrolled, and the mean age of the participants included in this study was 71.26 ± 7.1 years, including 7,170 (50. 58%) of the participants were males, 7,005 (49.42%) were females, and 5,482 participants (38.67%) were screened for cognitive decline. After PSM, logistic regression analysis revealed that multimorbidity was a risk factor for cognitive decline (OR = 1.392, 95% CI = 1.271–1.525, *p* < 0.001). The RCS show that the risk of cognitive decline is always greater in older adults with multimorbidity than in older adults without multimorbidity at the same age. Age, sex, marital status, educational level, monthly income, drinking status, participation in social activities, and exercise were influential factors for cognitive decline in older adults (*p* < 0.05). The incidence of cognitive decline in older adults with multimorbidity was also greater than that in older adults with one chronic disease (*p* < 0.001).

**Conclusion:**

The risk of cognitive decline in older adults with multimorbidity is greater than that in older adults without multimorbidity; therefore, the government should strengthen the prevention and treatment of multimorbidity in older adults to further protect their cognitive abilities.

## 1 Introduction

In recent years, global aging has become a serious problem, especially in China, where the health of older adult people has become an increasingly prominent issue. According to the National Bureau of Statistics of China, the population aged 60 years or older in China reached 280 million at the end of 2022, accounting for 19.8% of the country’s population (1). The population of older adults is affected by declining organismic functions, prolonged survival periods, and an increasing prevalence of incapacitated persons; one of the causes of these phenomena is the persistently high prevalence of multimorbidity among older people (2). Multimorbidity, a condition in which a person suffers from two or more chronic diseases or long-term medical conditions at the same time, has become a major public health problem that threatens the health status of older people, and previous studies have shown that the prevalence of multimorbidity in older adult people increased from 15.6 to 30.76% from 1998 to 2018 (3). In recent years, the prevalence of multimorbidity has ranged from 40 to 65% in different countries (2, 4, 5).

With the increasing age of the population, the incidence of cognitive dysfunction in older adult people is also increasing, with a prevalence rate of 15.5% (6). Cognitive ability, which refers to the ability of the human brain to process, store and extract information, is an important factor affecting the quality of life of older persons (7). Cognitive decline may affect the ability of older persons to perform daily living tasks (8). Older persons may face problems such as memory loss and slowed thinking, making it difficult for them to perform daily tasks on their own. It may also have a direct effect on the health of older adults, exacerbating their risk of developing dementia, including Alzheimer’s disease and other forms of dementia, which can further impair cognitive function in older adults, causing a vicious cycle (9, 10). Furthermore, family members often need more time and energy to care for older adults, placing a burden on society by increasing financial and emotional strain on the family (11). Cognitive decline in older adults is an important issue, and importantly, older adults are at risk for cognitive impairment and may not be able to cope with the effects of cognitive decline on their lives.

Many chronic diseases, such as diabetes, hypertension, respiratory diseases, digestive diseases, and degenerative osteoarthropathies, have been shown to affect cognitive function in older adults (12, 13). A study of multimorbidity combinations and the risk of developing dementia revealed that compared with people without multimorbidity, the risk of dementia was highest in multimorbidity clusters with cardiometabolic multimorbidity (mainly heart or circulatory diseases, hypercholesterolemia, stroke, diabetes, and high blood pressure) and mental health multimorbidity (mainly depression and other mood disorders), and lowest in inflammation and autoimmune clusters. The inflammation and autoimmune groups had intermediate levels of risk, and the cancer group had the lowest level of risk (14). However, the study was incomplete regarding controlling for confounders.

Many factors affect the cognitive ability of the older adult people, and some studies have shown that age, sex, mode of residence, marital status, educational level, and monthly income are among the multidimensional factors affecting multimorbidity and cognitive ability in older adult people, with age and sex taking the main role (15–19). Some lifestyles such as a sedentary lifestyle, smoking and diet are also risk factors for cognitive ability (20–22), the more unhealthy one’s lifestyle is, the greater the degree of cognitive decline (23). The above sociodemographic, health behavior, and lifestyle factors have an impact on cognitive performance in older adults, however, few studies have considered the impact of confounding factors on outcomes.

The propensity score matching (PSM) method developed by Rosenbaum and Rubin in 1983 (24) can be used to address nonrandomized data by using a single propensity score (PS) to represent multiple confounding variables, thus balancing the distribution of covariates between the multimorbidity and nonmultimorbidity groups, controlling for confounding bias between the two groups, and decreasing its interference with the results and improving accuracy in the results of the study of the effects of multimorbidity on cognitive decline. The restricted cubic spline (RCS) model can be used to visualizes the effect of small changes in variables on the results as a continuous curve and is an effective tool for exploring the differences between two sets of dependent variables. Therefore, the study further validated the effect of multimorbidity on cognitive ability in older adults by using the PSM to control for confounders and using the RCS to observe the difference in the risk of cognitive decline with age between older adults in the multimorbidity and nonmultimorbidity groups, with the aim of providing data to support the implementation of interventions for the prevention of cognitive decline in older adults.

## 2 Methods

### 2.1 Participants

Older adult people aged 60 years and older from seven medical institutions, three communities, and five nursing homes in Zunyi City, Guizhou Province, were selected for the study from October 2022 to September 2023 using convenience sampling. The inclusion criteria for the study subjects were as follows: (1) aged ≥60 years and (2) willing to voluntarily participate in this study and provide informed consent. The exclusion criteria were as follows: (1) had previous severe mental illness or severe cognitive impairment and were unable to communicate; (2) had acute critical illness (including shock, respiratory failure, acute heart failure, acute myocardial infarction, stroke, etc.) and were unable to cooperate with the survey; and (3) had acute exacerbation of chronic disease or terminal stage of disease (expected survival <3 months). This study was reviewed by the Ethics Review Committee of the Affiliated Hospital of Zunyi Medical University (KLL2022-814). The study was conducted in accordance with the principles outlined in the Declaration of Helsinki. Each participant voluntarily agreed to participate in the study and provided informed consent.

### 2.2 Sample size

Cognitive decline in older adult people is an important variable in this study, and by referring to a large amount of literature, we know that the rate of cognitive decline in China is approximately 28.6% (25), and the minimum required sample size of 7,845 was calculated using the following formula based on the 95% confidence interval (CI):


$$N=\frac{Z^{2}P\left(1-P\right)}{\delta^{2}}=\frac{1.96^{2}\times 0.286\times \left(1-0.286\right)}{0.01^{2}}\approx 7845$$

### 2.3 Assessment of cognitive function

The Mini-Mental State Examination (MMSE) was developed in 1975 by Folstein et al. (26) and has good reliability, with a Cronbach’s alpha coefficient of 0.916. The MMSE is used to measure cognitive function in older adults and consists of 30 items, including orientation (10 points), verbal ability (8 points), attention and calculation (5 points), delayed memory (3 points), immediate memory (3 points) and visuospatial skills (1 point). The total score ranges from 0 to 30 points, with higher scores indicating better cognitive functioning. Educational level, as a rank variable, is distinguished from the conditions of application of the MMSE as illiterate, elementary school, middle school and above. The MMSE defines cognitive decline on the basis of the following criteria: those who score < 17 at the illiteracy level, those who score < 20 at the elementary school level, and those who score < 24 at junior high school and above.

### 2.4 Assessment of multimorbidity

The 17 chronic diseases surveyed included hypertension, asthma, diabetes, heart disease, degenerative osteoarthropathy, cerebrovascular disease, hyperlipidemia, chronic lung disease, chronic bronchitis, cataracts, anxiety, renal disease, malignant tumors, liver disease, depression, gastrointestinal disease, and stomach disease. The total number of chronic diseases suffered by the respondents was counted, and multimorbidity was defined as respondents suffering from two or more of the above chronic diseases, whereas no multimorbidity was defined as respondents suffering from one or no chronic diseases.

### 2.5 Data collection

We selected age, sex, marital status, educational level, residence, mode of residence, type of medical insurance, monthly income, drinking status, smoking status, annual physical examination, participation in social activities, exercise, social support, and the presence of multimorbidity as covariates, with age as a continuous variable, and the other definitions are shown in Table 1.

**Table 1:** Definition of covariates.

Variable name	Definitions
Sex	1 = Male, 2 = Female
Marital status	1 = Married, 2 = Other (separated/divorced/widowed/unmarried)
Educational level	1 = Elementary school and below, 2 = Middle school, 3 = High school or middle school, 4 = College and above
Residence	1 = Urban, 2 = Rural
Mode of residence	1 = Not living alone, 2 = Living alone
Type of medical insurance	1 = Insurance, 2 = Self-funded
Monthly income (1 Yuan = 0.139 USD)	1 = ≥¥2000, 2 = <¥2000
Drinking	1 = Nondrinker, 2 = Drinker
Smoking	1 = Nonsmoker, 2 = Smoker
Annual physical examination	1 = Yes, 2 = No
Participation in social activities	1 = Yes, 2 = No
Exercise	1 = Yes, 2 = No
Social support	1 = Adequate, 2 = Lacking
Presence of multimorbidity	0 = No, 1 = Yes
Only one chronic disease	0 = No, 1 = Yes

The questionnaire was conducted in the form of face-to-face interview using a paper-based questionnaire. During the survey, the researcher asked the respondents questions and collected basic patient information and MMSE scores in a process that took approximately 30–40 min. The questionnaires were reviewed for completeness of content and retrieved in a timely manner at the end of the survey to ensure the accuracy of the survey results. The questionnaires completed on the day of the survey were uniformly checked.

### 2.6 Statistical analysis

To ensure the accuracy of the data, to prevent overall bias caused by too many or too few variables and to avoid data entry errors, outlier detection for continuous variables is needed. In this study, the box method was used to identify whether there were outliers, and the outliers were reconfirmed through the data source; if they were not available or confirmed as outliers, they were replaced with “null” to represent missing data. Then, the missing values were deleted.

SPSS 29.0 (IBM Inc., Armonk, NY, United States) and R 4.3.3 (R Foundation, Vienna, Austria) were used for statistical analysis. The quantitative data are presented as frequencies and percentages (%). The χ^2^-test was used to compare the differences between groups. In this study, after several attempts, the caliper value was set to 0.05, and the nearest neighbor matching method was used to match older adults with multimorbidity and older adults without multimorbidity at a ratio of 1:1, the standardized mean difference (SMD) of the covariates were used to assess the balance before and after PSM. After matching, conduct a univariate logistic regression analysis, considering cognitive decline as the dependent variable. Covariates and presence of multimorbidity as the independent variable. Then, incorporate variables that achieve statistical significance from the univariate regression into the multivariate logistic regression. Using the RCS model with age as the independent variable and cognitive decline as the outcome variable, the matched samples were categorized into a nonmultimorbidity group and a multimorbidity group to compare the difference in the risk of cognitive decline with increasing age between the two groups. The logistic regression analysis was done using SPSS 29.0 software and the RCS curves were done using R 4.3.3 software. *p* < 0.05 was considered to indicate statistical significance.

## 3 Results

### 3.1 Sample characteristics

A total of 14,175 respondents were enrolled, with an average age of 71.26 ± 7.19 years, including 7,170 (50.58%) males and 7,005 (49.42%) females; 12,110 (85.43%) were married, and 2065 (14.57%) were not married (separated/divorced/widowed/unmarried); 9,484 (66.91%) had an elementary school and below educational level, 2,818 (19.88%) had a junior high school educational level, 1,385 (9.77%) had a high school or junior college educational level, and 488 (3.44%) had a college educational level; and 6,921 (48.83%) were living in urban areas, 7,254 (51.17%) were living in rural areas, 13,387 (94.44%) were not living alone, and 788 (5.56%) were living alone. Multimorbidity was noted in 30.17% (4,276/14175) of respondents and 69.83% (9,899/14175) of respondents were without multimorbidity. Of these patients, 50.05% (4,954/9899) did not have chronic disease, and 49.95% (4,945/9899) had chronic disease.

### 3.2 Occurrence of cognitive decline in older adults

The incidence of cognitive decline in older adults with multimorbidity was 46.68% (1996/4276), which was significantly greater than that among older adults without multimorbidity (35.22%; 3486/9899) (*p* < 0.01) (Table 2). Among 9,221 older adults with chronic diseases, 3,908 (42.38%) experienced cognitive decline. Among the 9,221 older adult individuals with chronic diseases, 3,908 (42.38%) experienced cognitive decline, and the incidence of cognitive decline in older adult individuals with multimorbidity was 46.68% greater than that in older adult individuals with only one chronic disease (1912/4945) (38.67%). This difference was statistically significant (*p* < 0.05) (Table 3).

**Table 2:** Comparison of the incidence of cognitive decline in older adults with and without multimorbidity.

Group	Total (*n* = 14,175)	No cognitive decline (*n* = 8,693)	Cognitive decline (*n* = 5,482)	χ^2^	*P*
Presence of multimorbidity				165.453	<0.001
Yes	4,276	2,280 (53.32)	1996 (46.68)		
No	9,899	6,413 (64.78)	3,486 (35.22)		

χ^2^, chi-square test. *P* < 0.05: statistically significant.

**Table 3:** Comparison of the incidence of cognitive decline in older adults with multimorbidity versus those with only one chronic disease.

Group	Total (*n* = 9,221)	No cognitive decline (*n* = 5,313)	Cognitive decline (*n* = 3,908)	χ^2^	*P*
Multimorbidity versus only one chronic disease				60.307	<0.001
Multimorbidity	4,276	2,280(53.32)	1996(46.68)		
Only one chronic disease	4,945	3,033(61.33)	1912(38.67)		

χ^2^, chi-square test. *p* < 0.05: statistically significant.

### 3.3 Covariates and their relationships with cognitive decline in older adults with and without multimorbidity before and after PSM

#### 3.3.1 Comparison of the characteristics of older adults before and after PSM

Before PSM, among the 14,175 survey respondents, age, marital status, educational level, type of residence, mode of residence, monthly income, drinking status, smoking status, annual physical examination status, social activity status, and exercise status significantly differed (*p* < 0.05) between the older adults with no multimorbidity and the older adults with multimorbidity. After PSM, 4196 pairs of older adults were included, and the differences in the above variables were not statistically significant (*p* > 0.05) (Table 4). SMD analysis showed that all covariables were less than 0.1, suggesting that the results were well balanced (Supplementary Figure S1). Probability density estimation indicate that the two groups show adequate overlap after matching, with good control matching for each sample individual (Supplementary Figure S2).

**Table 4:** Covariates for older adults with and without multimorbidity before and after PSM.

Variable	Before PSM					After PSM				
	Total (n = 14,175)	Patients with no multimorbidity (*n* = 9,899)	Patients with multimorbidity (*n* = 4,276)	Z/χ^2^	*P*	Total (*n* = 8,392)	Patients with no multimorbidity (*n* = 4,196)	Patients with multimorbidity (*n* = 4,196)	Z/χ^2^	*P*
Age	71.00 (66.00, 76.00)	70.00 (65.00, 75.00)	72.00 (67.00, 78.00)	−14.438	<0.001	72.00 (67.00, 77.00)	72.00 (67.00, 78.00)	72.00 (67.00, 77.00)	−0.061	0.951
Sex				1.136	0.287				0.002	0.965
Male	7,170 (50.58)	4,978 (50.29)	2,192 (51.26)			4,259 (50.75)	2,117 (50.45)	2,142 (51.05)		
Female	7,005 (49.42)	4,921 (49.71)	2084 (48.74)			4,133 (49.25)	2079 (49.55)	2054 (48.95)		
Marital status				127.966	<0.001				0.064	0.8
Married	12,110 (85.43)	8,675 (87.64)	3,435 (80.33)			6,835 (81.45)	3,422 (81.55)	3,413 (81.34)		
Other (separated/divorced/widowed/unmarried)	2065 (14.57)	1,224 (12.36)	841 (19.67)			1,557 (18.55)	774 (18.45)	783 (18.66)		
Educational level				26.132	<0.001				4.471	0.215
Elementary school and below	9,484 (66.91)	6,738 (68.07)	2,746 (64.22)			5,487 (65.38)	2,782 (66.30)	2,705 (64.47)		
Middle school	2,818 (19.88)	1932 (19.52)	886 (20.72)			1,690 (20.14)	825 (19.66)	865 (20.61)		
High school or middle school	1,385 (9.77)	898 (9.07)	487 (11.39)			905 (10.78)	430 (10.25)	475 (11.32)		
College and above	488 (3.44)	331 (3.34)	157 (3.67)			310 (3.69)	159 (3.79)	151 (3.60)		
Residence				178.337	<0.001				0.415	0.519
Urban	7,254 (51.17)	4,701 (47.49)	2,553 (59.71)			4,979 (59.33)	2,504 (59.68)	2,475 (58.98)		
Rural	6,921 (48.83)	5,198 (52.51)	1723 (40.29)			3,413 (40.67)	1,692 (40.32)	1721 (41.02)		
Mode of residence				19.502	<0.001				0.646	0.422
Not living alone	13,387 (94.44)	9,404 (95.00)	3,983 (93.15)			7,856 (93.61)	3,937 (93.83)	3,919 (93.40)		
Living alone	788 (5.56)	495 (5.00)	293 (6.85)			536 (6.39)	259 (6.17)	277 (6.60)		
Type of medical insurance				2.541	0.111				3.053	0.081
Insurance	13,850 (97.71)	9,659 (97.58)	4,191 (98.01)			8,245 (98.25)	4,133 (98.50)	4,112 (98.00)		
Self-funded	325 (2.29)	240 (2.42)	85 (1.99)			147 (1.75)	63 (1.50)	84 (2.00)		
Monthly income				11.142	<0.001				0.375	0.54
≥¥2000	7,329 (51.7)	5,027 (50.78)	2,302 (53.84)			4,464 (53.19)	2,218 (52.86)	2,246 (53.53)		
<¥2000	6,846 (48.3)	4,872 (49.22)	1974 (46.16)			3,928 (46.81)	1978 (47.14)	1950 (46.47)		
Drinking				28.586	<0.001				0.153	0.696
Nondrinker	12,356 (87.17)	8,531 (86.18)	3,825 (89.45)			7,505 (89.43)	3,758 (89.56)	3,747 (89.30)		
Drinker	1819 (12.83)	1,368 (13.82)	451 (10.55)			887 (10.57)	438 (10.44)	449 (10.70)		
Smoking				12.808	<0.001				0.127	0.722
Nonsmoker	11,684 (82.43)	8,085 (81.67)	3,599 (84.17)			7,038 (83.87)	3,513 (83.72)	3,525 (84.01)		
Smoker	2,491 (17.57)	1814 (18.33)	677 (15.83)			1,354 (16.13)	683 (16.28)	671 (15.99)		
Annual physical examination				129.385	<0.001				0.382	0.537
Yes	7,128 (50.29)	4,667 (47.15)	2,461 (57.55)			4,816 (57.39)	2,422 (57.72)	2,394 (57.05)		
No	7,047 (49.71)	5,232 (52.85)	1815 (42.45)			3,576 (42.61)	1774 (42.28)	1802 (42.95)		
Participation in social activities				109.679	<0.001				0.598	0.44
Yes	8,906 (62.83)	6,496 (65.62)	2,410 (56.36)			4,831 (57.57)	2,433 (57.98)	2,398 (57.15)		
No	5,269 (37.17)	3,403 (34.38)	1866 (43.64)			3,561 (42.43)	1763 (42.02)	1798 (42.85)		
Exercise				5.979	0.014				0.736	0.391
Yes	10,774 (76.01)	7,581 (76.58)	3,193 (74.67)			6,298 (75.05)	3,166 (75.45)	3,132 (74.64)		
No	3,401 (23.99)	2,318 (23.42)	1,083 (25.33)			2094 (24.95)	1,030 (24.55)	1,064 (25.36)		
Social support				2.096	0.148				2.078	0.149
Adequate	12,655 (89.28)	8,862 (89.52)	3,793 (88.70)			7,485 (89.19)	3,763 (89.68)	3,722 (88.70)		
Lacking	1,520 (10.72)	1,037 (10.48)	483 (11.30)			907 (10.81)	433 (10.32)	474 (11.30)		

PSM, propensity score matching; χ^2^, chi-square test. *P* < 0.05: statistically significant.

#### 3.3.2 Cognitive decline in older adults with and without multimorbidity after PSM

After PSM, the incidence of cognitive decline in older adults with multimorbidity was 46.16%, which was significantly greater than that in older adults without multimorbidity (38.89%; *p* < 0.05). The differences in the incidence of cognitive decline among older adults of different ages, sexes, marital status, educational level, residence, mode of residence, monthly income, drinking, smoking, social activities, and exercise were statistically significant (*p* < 0.05) (Table 5).

**Table 5:** Differential covariate comparison of the occurrence of cognitive decline in older adults after PSM.

Variable	Total (*n* = 8,392)	No cognitive decline (*n* = 4,823)	Cognitive decline (*n* = 3,569)	Z/χ^2^	*P*
Age	72.00(67.00–77.00)	70.00(66.00–75.50)	74.00(69.00–80.00)	20.568	<0.001
Sex				69.156	<0.001
Male	4,259(50.75)	2,636(54.65)	1,623(45.47)		
Female	4,133(49.25)	2,187(45.35)	1946(54.53)		
Marital status				78.344	<0.001
Married	6,835(81.45)	4,084(84.68)	2,751(77.08)		
Others (separated/divorced/widowed/unmarried)	1,557(18.55)	739(15.32)	818(22.92)		
Educational level				110.870	<0.001
Elementary school and below	5,487(65.38)	3,073(63.72)	2,414(67.64)		
Middle school	1,690(20.14)	895(18.56)	795(22.28)		
High school or middle school	905(10.78)	614(12.73)	291(8.15)		
College and above	310(3.69)	241(5.00)	69(1.93)		
Residence				10.042	0.002
Urban	4,979(59.33)	2,932(60.79)	2047(57.36)		
Rural	3,413(40.67)	1891(39.21)	1,522(42.64)		
Mode of residence				9.452	0.002
Not living alone	7,856(93.61)	4,549(94.32)	3,307(92.66)		
Living alone	536(6.39)	274(5.68)	262(7.34)		
Type of medical insurance				3.735	0.053
Insurance	8,245(98.25)	4,750(98.49)	3,495(97.93)		
Self-funded	147(1.75)	73(1.51)	74(2.07)		
Monthly income				115.127	<0.001
≥¥2000	4,464(53.19)	2,808(58.22)	1,656(46.40)		
<¥2000	3,928(46.81)	2015(41.78)	1913(53.60)		
Drinking				47.760	<0.001
Nondrinker	7,505(89.43)	4,217(87.44)	3,288(92.13)		
Drinker	887(10.57)	606(12.56)	281(7.87)		
Smoking				43.467	<0.001
Nonsmoker	7,038(83.87)	3,935(81.59)	3,103(86.94)		
Smoker	1,354(16.13)	888(18.41)	466(13.06)		
Annual physical examination				3.717	0.054
Yes	4,816(57.39)	2,811(58.28)	2005(56.18)		
No	3,576(42.61)	2012(41.72)	1,564(43.82)		
Participation in social activities				142.875	<0.001
Yes	4,831(57.57)	3,044(63.11)	1787(50.07)		
No	3,561(42.43)	1779(36.89)	1782(49.93)		
Exercise				16.851	<0.001
Yes	6,298(75.05)	3,700(76.72)	2,598(72.79)		
No	2094(24.95)	1,123(23.28)	971(27.21)		
Social support				0.092	0.762
Adequate	7,485(89.19)	4,306(89.28)	3,179(89.07)		
Lacking	907(10.81)	517(10.72)	390(10.93)		
Presence of multimorbidity				45.353	<0.001
Yes	4,196(50.00)	2,259(46.84)	1937(54.27)		
No	4,196(50.00)	2,564(53.16)	1,632(45.73)		

χ^2^, chi-square test. *P* < 0.05: statistically significant.

#### 3.3.3 Multivariate logistic regression analysis of cognitive decline in older adults

**Figure 1:**
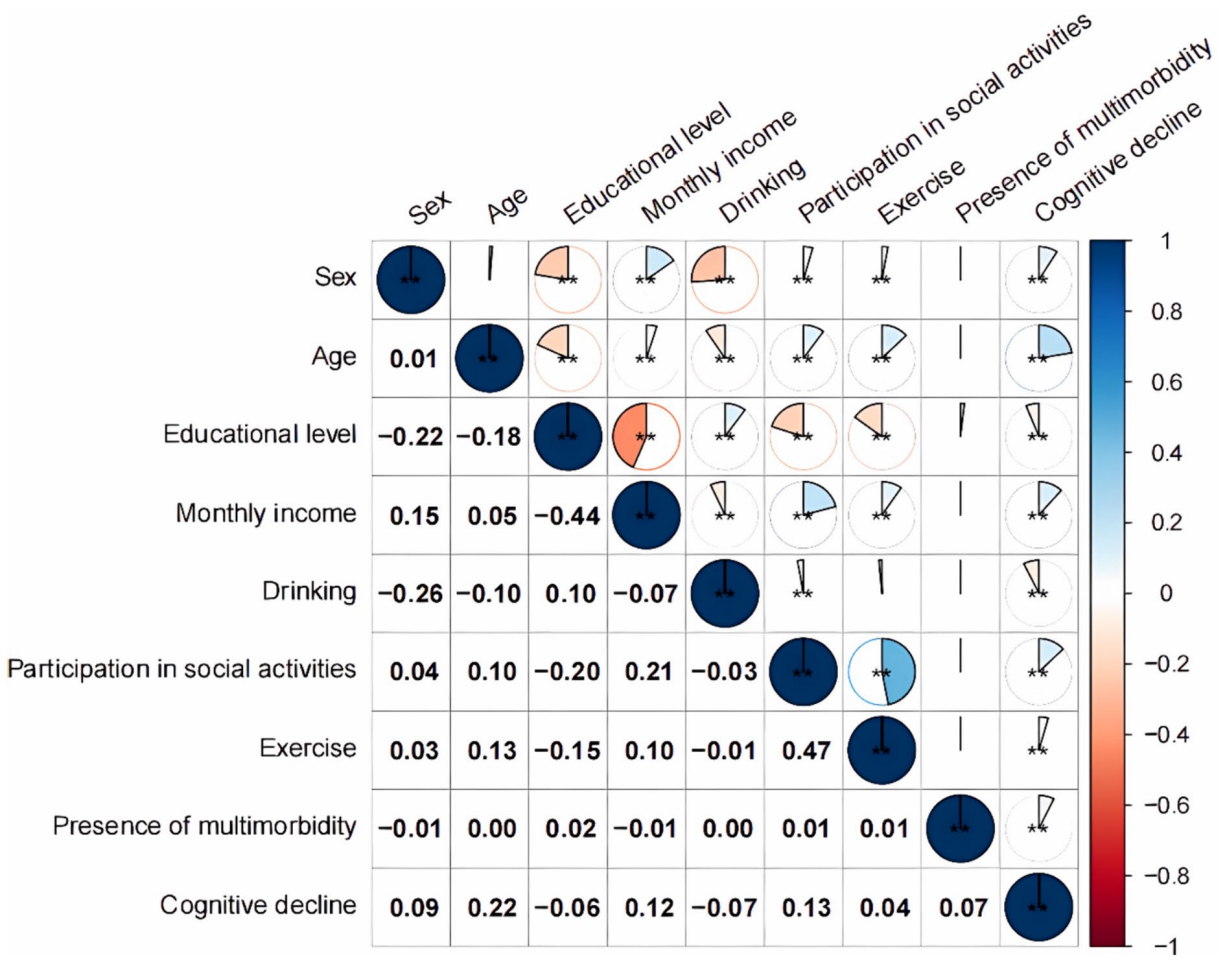
Spearman correlation among independent variables. **p < 0.01 (two-tailed).

Univariate logistic regression analyses with the presence of cognitive decline as the dependent variable and the included indicators as independent variables (see Table 1 for defined values), incorporated variables that were statistically significant in univariate regression into multivariate regression analysis, showed that multimorbidity was a risk factor for the emergence of cognitive decline in older adult people [OR = 1.392, 95% CI (1.271, 1.525), *p* < 0.001]. Moreover, age, sex, educational level, monthly income, drinking status, participation in social activities, and physical exercise were also influential factors for the development of cognitive decline in older adults (*p* < 0.05), and no significant relationship was noted with cognitive decline at the high school or secondary school level (*p* > 0.05) (Table 6). According to the correlation analysis in Figure 1, a significant difference existed (*p* < 0.001) except between sex and age, between exercise and drinking, and between the presence of multimorbidity and the individual variables (*p* > 0.05).

**Table 6:** Univariate and multivariate logistic regression analyses of factors influencing cognitive ability after PSM.

	Univariate logistic regression analyses					Multivariate logistic regression analyses				
Variable	*β*	*SE*	*P*	*OR*	*95% CI*	*β*	*SE*	*P*	*OR*	*95% CI*
Age	0.064	0.003	<0.001	1.066	(1.060, 1.073)	0.065	0.003	<0.001	1.067	(1.060, 1.075)
Sex (reference male)										
Female	0.368	0.044	<0.001	1.455	(1.325, 1.576)	0.299	0.052	<0.001	1.349	(1.218, 1.495)
Marital status (reference married)										
Other (separated/divorced/widowed/unmarried)	0.497	0.056	<0.001	1.643	(1.471, 1.835)	0.104	0.067	0.120	1.11	(0.973, 1.266)
Educational level (reference elementary school and below)										
Middle school	0.123	0.056	0.028	1.131	(1.014, 1.261)	0.612	0.065	<0.001	1.844	(1.624, 2.095)
High school or middle school	−0.505	0.076	<0.001	0.603	(0.520, 0.700)	0.035	0.087	0.688	1.035	(0.873, 1.228)
College and above	−1.009	0.139	<0.001	0.364	(0.277, 0.479)	−0.382	0.151	0.011	0.682	(0.508, 0.917)
Type of residence (reference urban)										
Rural	0.142	0.045	0.002	1.153	(1.056, 1.259)	0.013	0.053	0.811	1.013	(0.913, 1.123)
Mode of residence (reference not living alone)										
Living alone	0.274	0.089	0.002	1.315	(1.104, 1.567)	−0.009	0.102	0.929	0.991	(0.811, 1.210)
Type of medical insurance (reference insurance)										
Self-funded	0.32	0.166	0.054	1.378	(0.994, 1.909)					
Monthly income (reference≥￥2000)										
<￥2000	0.476	0.044	<0.001	1.61	(1.475, 1.756)	0.418	0.053	<0.001	1.519	(1.368, 1.686)
Drinking (reference nondrinker)										
Drinker	−0.52	0.076	<0.001	0.595	(0.513, 0.690)	−0.21	0.088	0.017	0.811	(0.682, 0.963)
Smoking (reference nonsmoker)										
Smoker	−0.407	0.062	<0.001	0.665	(0.589, 0.752)	−0.11	0.075	0.142	0.895	(0.773, 1.038)
Annual physical examination (reference “yes”)										
No	0.086	0.045	0.054	1.09	(0.999, 1.189)					
Participation in social activities (reference “yes”)										
No	0.534	0.045	<0.001	1.706	(1.563, 1.863)	0.503	0.054	<0.001	1.654	(1.488, 1.840)
Exercise (reference “yes”)										
No	0.208	0.051	<0.001	1.231	(1.115, 1.360)	−0.218	0.061	<0.001	0.804	(0.713, 0.907)
Social support (reference adequate)										
Lacking	0.022	0.071	0.762	1.022	(0.889, 1.174)					
Presence of multimorbidity (reference no multimorbidity)										
Multimorbidity	0.298	0.044	<0.001	1.347	(1.235, 1.469)	0.331	0.047	<0.001	1.392	(1.271, 1.525)

β, Standardized regression coefficient; SE, Standard error; OR, Odds ratio; CI, Confidence interval; χ^2^, Chi-square test. *P* < 0.05: statistically significant.

#### 3.3.4 Comparison of differences in dose–response relationships between age and the risk of cognitive decline in older adults with and without multimorbidity

**Figure 2:**
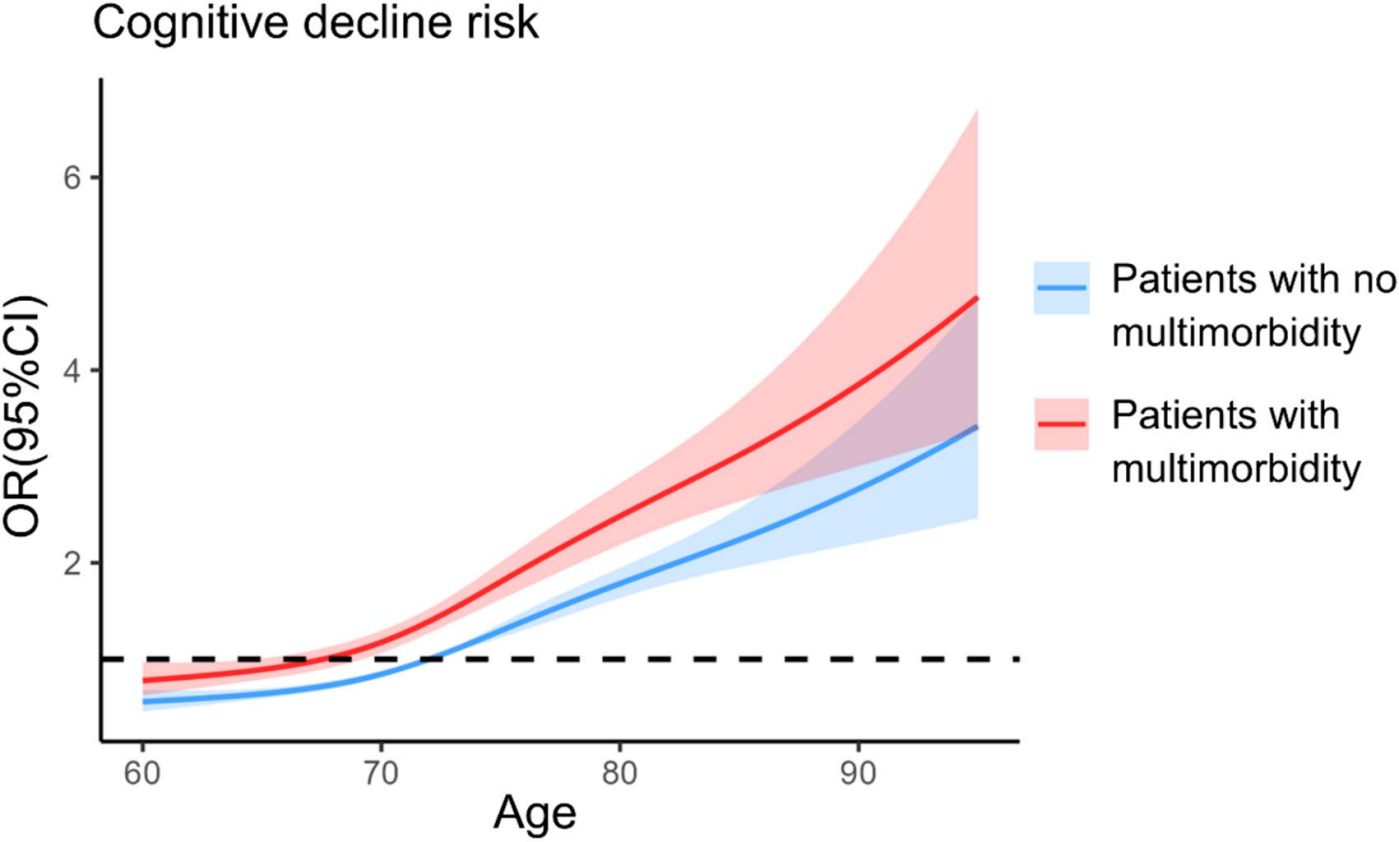
Analysis of restricted cubic spline regression. The model was based on logistic regression models and adjusted for sex, marital status, educational level, residence, mode of residence, type of medical insurance, monthly income, drinking, smoking, annual physical examination, participation in social activities, exercise, social support, and presence of multimorbidity.

The relationship curve between age and cognitive decline was plotted for both groups of older adults using quartiles of age as nodes and OR at median age in the nonmultimorbidity group as a reference line. After adjusting for all covariates in the model, the RCS curves revealed a significant dose–response relationship between age and the risk of cognitive decline (overall *p* < 0.001), with the risk of cognitive decline being significantly greater in the multimorbidity group than in the nonmultimorbidity group at the same age (Figure 2). A positive correlation was noted between age and the risk of cognitive decline in the nonmultimorbidity group (*P*_Nonlinear_ = 0.030), and *P*_Nonlinear_ = 0.342 in the multimorbidity group.

### 3.4 Patients with multimorbidity and older adults with one chronic disease before and after PSM and their cognitive decline

PSM was conducted on 9,233 older adults with chronic diseases. Before PSM, statistically significant differences (*p* < 0.05) in age, marital status, residence, mode of residence, drinking status, smoking status, annual physical examination, participation in social activities, and exercise were noted between older adults with multimorbidity and older adults with one chronic disease. After PSM, for 3,933 pairs of older adults, the above variables were not significantly different (*p* > 0.05) (Table 7).

**Table 7:** Comparison of general conditions between older adults with multimorbidity and those with only one chronic disease before and after PSM.

Variable	Before PSM					After PSM				
	Total (*n* = 9,221)	Multimorbidity (*n* = 4,276)	Only one chronic disease (*n* = 4,945)	Z/χ^2^	*P*	Total (*n* = 7,866)	Multimorbidity (*n* = 3,933)	Only one chronic disease (*n* = 3,933)	Z/χ^2^	*P*
Age	71.00 (66.00, 77.00)	72.00 (67.00, 78.00)	70.00 (66.00, 76.00)	−8.871	<0.001	71.00 (67.00, 77.00)	71.00 (67.00, 77.00)	71.00 (67.00, 77.00)	−0.787	0.431
Sex				0.314	0.575				0.269	0.604
Male	4,698 (50.95)	2,192 (51.26)	2,506 (50.68)			4,037 (51.32)	2007 (51.03)	2030 (51.61)		
Female	4,523 (49.05)	2084 (48.74)	2,439 (49.32)			3,829 (48.68)	1926 (48.97)	1903 (48.39)		
Marital status				51.245	<0.001				0.237	0.626
Married	7,683 (83.32)	3,435 (80.33)	4,248 (85.90)			6,576 (83.6)	3,280 (83.40)	3,296 (83.80)		
Other (separated/divorced/widowed/unmarried)	1,538 (16.68)	841 (19.67)	697 (14.10)			1,290 (16.4)	653 (16.60)	637 (16.20)		
Educational level				7.715	0.052				0.430	0.934
Elementary school and below	6,034 (65.44)	2,746 (64.22)	3,288 (66.49)			5,098 (64.81)	2,555 (64.96)	2,543 (64.66)		
Middle school	1887 (20.46)	886 (20.72)	1,001 (20.24)			1,627 (20.68)	810 (20.59)	817 (20.77)		
High school or middle school	973 (10.55)	487 (11.39)	486 (9.83)			849 (10.79)	427 (10.86)	422 (10.73)		
College and above	327 (3.55)	157 (3.67)	170 (3.44)			292 (3.71)	141 (3.59)	151 (3.84)		
Residence				52.705	<0.001				0.002	0.964
Urban	5,133 (55.67)	2,553 (59.71)	2,580 (52.17)			4,500 (57.21)	2,251 (57.23)	2,249 (57.18)		
Rural	4,088 (44.33)	1723 (40.29)	2,365 (47.83)			3,366 (42.79)	1,682 (42.77)	1,684 (42.82)		
Mode of residence				11.793	<0.001				0.385	0.535
Not living alone	8,673 (94.06)	3,983 (93.15)	4,690 (94.84)			7,399 (94.06)	3,706 (94.23)	3,693 (93.90)		
Living alone	548 (5.94)	293 (6.85)	255 (5.16)			467 (5.94)	227 (5.77)	240 (6.10)		
Type of medical insurance				2.213	0.137				0.025	0.874
Insurance	9,015 (97.77)	4,191 (98.01)	4,824 (97.55)			7,704 (97.94)	3,853 (97.97)	3,851 (97.92)		
Self-funded	206 (2.23)	85 (1.99)	121 (2.45)			162 (2.06)	80 (2.03)	82 (2.08)		
Monthly income				3.479	0.062				0.319	0.572
≥¥2000	4,868 (52.79)	2,302 (53.84)	2,566 (51.89)			4,167 (52.97)	2071 (52.66)	2096 (53.29)		
<¥2000	4,353 (47.21)	1974 (46.16)	2,379 (48.11)			3,699 (47.03)	1862 (47.34)	1837 (46.71)		
Drinking				12.610	<0.001				0.032	0.858
Nondrinker	8,130 (88.17)	3,825 (89.45)	4,305 (87.06)			6,981 (88.75)	3,493 (88.81)	3,488 (88.69)		
Drinker	1,091 (11.83)	451 (10.55)	640 (12.94)			885 (11.25)	440 (11.19)	445 (11.31)		
Smoking				7.485	0.006				0.008	0.927
Nonsmoker	7,655 (83.02)	3,599 (84.17)	4,056 (82.02)			6,575 (83.59)	3,286 (83.55)	3,289 (83.63)		
Smoker	1,566 (16.98)	677 (15.83)	889 (17.98)			1,291 (16.41)	647 (16.45)	644 (16.37)		
Annual physical examination				27.588	<0.001				0.186	0.666
Yes	5,037 (54.63)	2,461 (57.55)	2,576 (52.09)			4,387 (55.77)	2,203 (56.01)	2,184 (55.53)		
No	4,184 (45.37)	1815 (42.45)	2,369 (47.91)			3,479 (44.23)	1730 (43.99)	1749 (44.47)		
Participation in social activities				45.442	<0.001				0.089	0.766
Yes	5,538 (60.06)	2,410 (56.36)	3,128 (63.26)			4,635 (58.92)	2,324 (59.09)	2,311 (58.76)		
No	3,683 (39.94)	1866 (43.64)	1817 (36.74)			3,231 (41.08)	1,609 (40.91)	1,622 (41.24)		
Exercise				4.844	0.028				0.196	0.658
Yes	6,983 (75.73)	3,193 (74.67)	3,790 (76.64)			5,907 (75.1)	2,962 (75.31)	2,945 (74.88)		
No	2,238 (24.27)	1,083 (25.33)	1,155 (23.36)			1959 (24.9)	971 (24.69)	988 (25.12)		
Social support				0.000	0.989				0.178	0.673
Adequate	8,179 (88.7)	3,793 (88.70)	4,386 (88.70)			6,950 (88.35)	3,481 (88.51)	3,469 (88.20)		
Lacking	1,042 (11.3)	483 (11.30)	559 (11.30)			916 (11.65)	452 (11.49)	464 (11.80)		

PSM, propensity score matching; χ^2^, chi-square test. *P* < 0.05: statistically significant.

The results revealed that the incidence of cognitive decline in older adults with multimorbidity was 45.64%, which was significantly greater than that the 39.69% reported in older adults with only one chronic disease (*p* < 0.05) (Table 8).

**Table 8:** Incidence of cognitive decline in older adults with multimorbidity versus one chronic disease after PSM.

Group	Total (*n* = 7,866)	No cognitive decline (*n* = 4,510)	Cognitive decline (*n* = 3,356)	χ^2^	*P*
Multimorbidity versus having a chronic disease				28.457	<0.001
Multimorbidity	3,933	2,138(54.36)	1795(45.64)		
Only one chronic disease	3,933	2,372(60.31)	1,561(39.69)		

χ^2^, chi-square test. *P* < 0.05: statistically significant.

## 4 Discussion

In this study, we used PSM to balance confounding bias between older adults with and without multimorbidity to ensure the comparability of the observation and control groups and the reliability of the findings. Therefore, we further validated the effect of multimorbidity on cognitive performance in older adults. We conclude that the incidence rate of cognitive decline in older adults with multimorbidity was 1.392 times greater than that in older adults without multimorbidity. Similarly, the RCS curves revealed that older adults with multimorbidity are at greater risk of cognitive decline than older adults without multimorbidity at the same age.

### 4.1 Increased incidence of cognitive decline in older adults

A total of 14,175 survey respondents were included, 38.67% of whom experienced cognitive decline, which was similar to the findings of Cong Lin (27) (36.02%). This percentage was higher than that reported by Lei Shen (28) for urban older adults (16.2%) but slightly lower than that reported by Wang et al. (29) for rural older adults in North China (42.9%). This difference was likely attributed to the relative scarcity of health care resources for older adults in rural areas compared with those in urban areas, resulting in the possibility of older adults not being able to receive adequate health care services, such as early detection and treatment (30). This percentage was slightly greater than that reported in the Li et al. (31) survey of older adults ≥65 years old (30.52%) likely because the age of the respondents was slightly greater than that of the older adults (>60 years old) selected for this study. In previous studies, Hou et al. (32) collected data on older adults from 2013 to 2017 and reported a cognitive decline incidence of 16.8%, and Ding et al. (33) surveyed community residents in 2009 and reported a cognitive decline incidence of 20.1%. Although the incidence of cognitive decline in older adults has not been consistent across different studies, generally, the incidence of cognitive decline in Chinese older adult individuals is gradually increasing, probably due to increasing life expectancy and aging of the population (2, 34). The rate of cognitive decline accelerates with age (35). These findings are also consistent with the results of this study. As the population ages, the incidence of cognitive decline in older adults increases, indicating the need for early intervention programs.

### 4.2 Multimorbidity as a risk factor for developing cognitive decline in older adults

The results of this study revealed that the incidence of cognitive decline in older adults with multimorbidity and in those with no multimorbidity after PSM was as high as 46.16%, and the risk of developing cognitive decline in older adults with multimorbidity was 1.392 times greater than that in older adults with no multimorbidity. The RCS curves provide more intuitive evidence that the risk of cognitive decline is higher in older adults with multimorbidity after controlling for age factors. After PSM, in older adults with multimorbidity versus older adults with only one chronic disease, the incidence of cognitive decline in older adults with multimorbidity was 45.64%, which significantly differed from that in older adults with only one chronic disease (39.69%), indicating that multimorbidity is a risk factor for developing cognitive decline in older adults. This finding is consistent with the results of previous studies (36, 37), and it can be analyzed from the perspectives of medication effects, secondary effects of chronic diseases, and chronic disease characteristics. The number and type of medications taken by patients with multimorbidity, multiple medication regimens and drug–drug interactions or cumulative effects may increase the risk of cognitive decline in patients with multimorbidity (36, 38). In addition, multimorbidity may lead to a decrease in the independence of older adults’ related functions, which reduces the number of social activities (31, 39).

Furthermore, the underlying biological mechanisms of multimorbidity and cognitive decline are largely unknown, but several possible effects of chronic disease on cognitive performance which underlie this unknown mechanism. It has been found that chronic inflammation, increased frequency of cerebrovascular disease, and inadequate cerebral oxygenation may increase the risk of developing cognitive decline in patients with multimorbidity (40). Chronic diseases are often associated with low-grade chronic inflammation, which plays an important role in the pathogenesis of cardiometabolic multimorbidity, and inflammation and hereditary predisposition further increase the risk of cognitive decline induced by multimorbidity (41). In cardiovascular-metabolic multimorbidity, hypertension alters cerebral blood flow, which may damage blood vessels in the brain and white matter (causing white matter hyperintensity), leading to a decrease in overall brain volume due to the damage to neurons and their connections and neuronal death, which in turn leads to cognitive decline (42). When an individual is in a chronic hypertensive state, vasoconstriction reduces vascular elasticity and increases the risk of cerebral hemorrhage, cerebral infarction, and cerebral small-vessel disease, which are all known risk factors for cognitive decline (43). Diabetes disrupts neuronal glucose metabolism and increases oxidative stress, and aggravated cerebrovascular damage leads to decreased cerebral blood flow and oxygen supply, impairing cognitive function (44). Neuroinflammation, a result of systemic inflammation in diabetes, can lead to synaptic dysfunction and neuronal loss, which in turn increases the risk of cognitive decline (18, 22, 45). There are also studies on the relationship between genes and cognitive function that show that five genes, PFKFB4, PDK3, KIAA0319L, CEBPD, and PHC2T, have the potential to recognize Alzheimer’s disease (46).

However, the current clinical model is mostly a single-disease management model, and a standard multimorbidity management model that can meet the health management needs of older adult individuals with chronic diseases has not yet been developed (47). Medical institutions and health centers should fully recognize the severe damage caused by multimorbidity to the cognitive ability of older adult people and should pay attention not only to the recovery of physical health but also to cognitive function abnormalities.

### 4.3 Factors influencing cognitive decline in older adults

Age, sex, low monthly income, and lack of socialization are risk factors for developing cognitive decline in older adults. This study revealed that the risk of cognitive decline increased by 1.067 times with each year of age compared with that in the previous year, and the risk of cognitive decline increased with age (35). Older women have a greater rate of cognitive decline; consistent with the findings of previous studies, the prevalence of cognitive impairment in women is significantly greater than that in men globally (48). This difference arises as a result of a combination of physiological factors (i.e., differences in the levels of sex hormones at the cellular, organ, and systemic levels) and social factors (environmental, social, cultural, and other factors) (49, 50). Older adults with a high monthly income can afford a higher-quality diet and lifestyle, with access to more adequate nutrition and better medical care, which, consistent with the results of the present study, helps maintain their cognitive function (51, 52). Psychosocial factors are important factors affecting cognitive performance in older adults. Negative emotions increase the risk of cognitive decline in older adults (39). Older adults who frequently participate in social activities usually have stronger social support and emotional attachments.

In addition to the above risk factors, the results of this study revealed that a high education level, alcohol consumption and not engaging in exercise were protective factors against cognitive decline in older adults. The higher the education level of older adults was, the lower the prevalence of cognitive decline was, which is in line with the findings of previous studies (53, 54). Older adults with a high educational level usually have a greater knowledge base and stronger self-management skills (30, 55), moreover, they are more adaptable to new technologies and information, which allows them to remain socially connected, gain more information, in turn, delay cognitive decline (55). The prevalence of cognitive decline in older adults who consume alcohol is 0.758 times greater than that in older adults who do not drink alcohol, and older adults who do not drink alcohol are more likely to experience cognitive decline, which is not entirely consistent with the results of previous studies (29, 56). This study suggested that light alcohol consumption may have a protective effect on cognitive function in older adults, whereas heavy alcohol consumption adversely affects cognitive function. A cohort study in the United States (57) also demonstrated that light or moderate alcohol consumption (women less than 8 drinks per week and men less than 15 drinks per week) was positively associated with cognitive improvement in middle-aged and older adults. A recent study of more than 3.93 million people (58) showed that light and moderate drinkers had a reduced risk of dementia compared with nondrinkers, but heavy drinkers had an 8% increased risk of dementia. There is still much controversy about the association between moderate drinking and cognition, and the present study did not grade the level of alcohol consumption, which could be improved accordingly in future studies. Although most studies consider exercise as a protective factor for cognitive decline, the results of the present study revealed that the incidence of cognitive decline in older adult people who do not exercise is 0.825 times higher than that of older adult people who exercise, a result that is shared by only a few studies. The risk of cognitive decline was greater in older adults who exercised in the present study, and an intervention study of older adults who engaged in physical activity for 18 months revealed no significant improvement in cognitive function in older adults (59). A systematic evaluation and meta-analysis (60) revealed a very small correlation between exercise and the risk of experiencing cognitive decline, with no significant dose–response association. The correlation coefficient between exercise and cognition in this study was 0.04, which is a very weak correlation.

On the basis of the results of this study, the government can optimize the allocation of medical resources, increase attention and investment in patients with multimorbidity and those at potential risk, achieve early identification of cognitive decline in older adults at risk of multimorbidity, and ensure the provision of timely and effective medical services and health management. Public health promotion and education campaigns can underscore the link between multimorbidity and cognitive decline, along with the aforementioned influencing factors, and increase the public’s awareness of self-care and health management skills, especially among older adult individuals.

Our research has two main strengths. Observational studies often involve multiple covariates and complex data structures, and PSM facilitates the elimination of selection bias, reduces the effect of potential confounders, and improves between-group balance, which greatly improves the reliability of the results. The above results and discussion of the influencing factors in this study provide new ideas about the factors of cognitive functioning.

This study has several limitations. First, this study used patient self-reported data, which may exhibit some bias. In addition, the cross-sectional study design limited the ability to infer causality. Although several confounders were controlled for in this study, potential factors that affect cognitive function in older adults with multimorbidity remain unaddressed. The results of a meta-analysis revealed that abnormal sleep duration and insomnia were associated with increased odds of multimorbidity (61). Adequate and high-quality sleep is essential for memory consolidation and the clearance of metabolic waste from the brain (62). Insufficient or poor-quality sleep is closely related to cognitive decline, potentially leading to reduced attention, memory impairment, and decreased decision-making ability (63). Adequate nutrition is even more known to reduce the risk of cognitive decline and promote brain health while influencing the incidence of various chronic diseases, specifically, live microbes, vegetables, fish, and nuts, is believed to promote brain health (16, 64). Future studies could incorporate additional confounders to deepen the understanding of the relationship between multimorbidity and cognitive decline.

## 5 Conclusion

In conclusion, the increased risk of cognitive decline in older adults with multimorbidity was further validated in this study. After controlling for confounders using PSM, RCS curves were used to demonstrate a greater risk of cognitive decline in older adults with multimorbidity at the same age. And factors that increase the risk of cognitive decline in older adults were identified, including advanced age, female sex, bottom monthly income, and lack of socialization. The protective factors for cognitive decline in older adults were a high education level, alcohol consumption, and no physical activity. These findings suggest that the prevention and treatment of chronic diseases in older adult individuals should be strengthened and that we should also focus on early intervention for individuals with cognitive decline. In the future, the in-depth association between multimorbidity and cognitive ability should be investigated by controlling for confounders and expanding the survey sample size.

## Data Availability

The original data supporting the conclusions of this article will be provided by the authors, further inquiries can be directed to the corresponding author.

## Supplementary material

Supplementary Figure S1 Absolute value of SMD for covariates.

Supplementary Figure S2 Probability density estimation diagram.
