# Supplementary figures and images for: The relationship between multimorbidity and cognitive function in older Chinese adults: based on propensity score matching
Source: Front Public Health. 2024 Sep 12;12:1422000. doi: 10.3389/fpubh.2024.1422000 (PMC11425792; doi:10.3389/fpubh.2024.1422000)

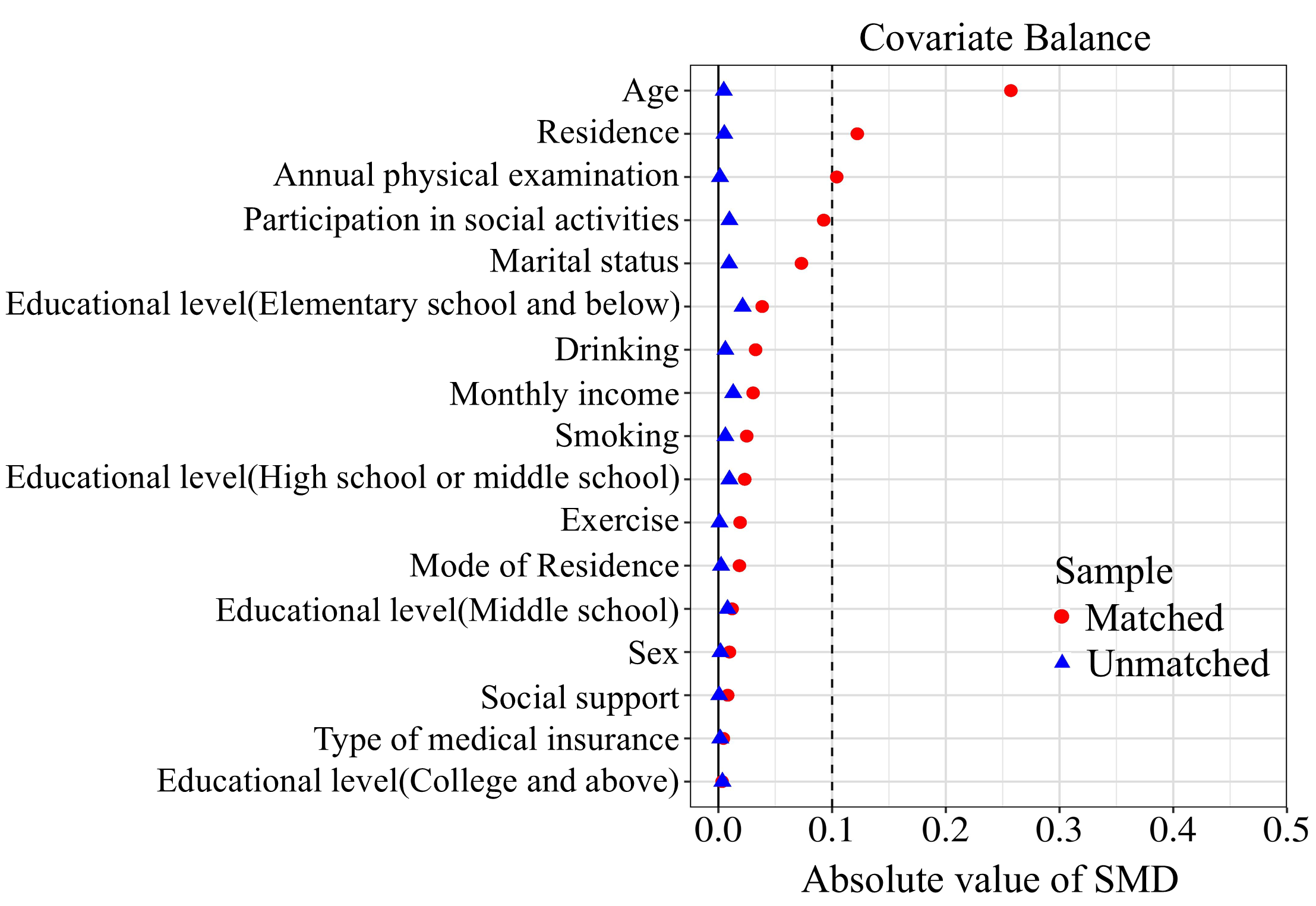

Supplement: Supplementary Figure S1 — Absolute value of SMD for covariates. [file Image_1.JPEG]

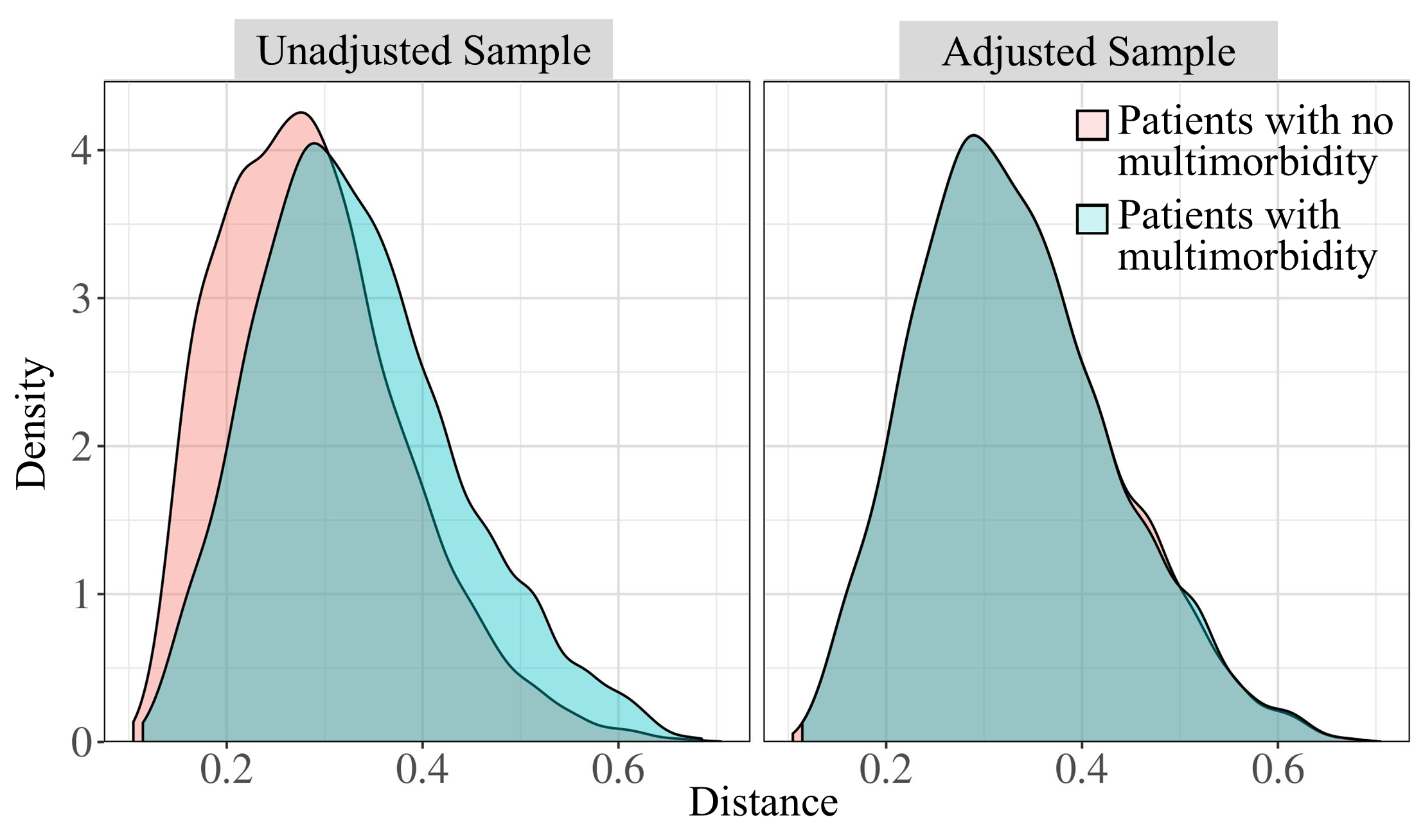

Supplement: Supplementary Figure S2 — Probability density estimation diagram. [file Image_2.JPEG]
